# Elderly Patients in a Large Nephrology Unit: Who Are Our Old, Old-Old and Oldest-Old Patients?

**DOI:** 10.3390/jcm10061168

**Published:** 2021-03-11

**Authors:** Massimo Torreggiani, Antoine Chatrenet, Antioco Fois, Maria Rita Moio, Béatrice Mazé, Jean Philippe Coindre, Romain Crochette, Mickael Sigogne, Samuel Wacrenier, Léna Lecointre, Conrad Breuer, Hafedh Fessi, Giorgina Barbara Piccoli

**Affiliations:** 1 Néphrologie et Dialyse, Centre Hospitalier Le Mans, 194 Avenue Rubillard, 72037 Le Mans, France; mtorreggiani@ch-lemans.fr (M.T.); achatrenet@ch-lemans.fr (A.C.); afois@ch-lemans.fr (A.F.); mariaritamoio@gmail.com (M.R.M.); bmaze@ch-lemans.fr (B.M.); jpcoindre@ch-lemans.fr (J.P.C.); rcrochette@ch-lemans.fr (R.C.); msigogne@ch-lemans.fr (M.S.); swacrenier@ch-lemans.fr (S.W.); 2 Direction, Centre Hospitalier Le Mans, Avenue Roubillard 194, 72037 Le Mans, France; llecointre@ch-lemans.fr (L.L.); cbreuer@ch-lemans.fr (C.B.); 3 Department of Nephrology, Hospital Tenon, 75020 Paris, France; hafedh.fessi@aphp.fr

**Keywords:** chronic kidney disease, elderly, CKD-EPI, kidney function, equation

## Abstract

The world population is aging, and the prevalence of chronic kidney disease (CKD) is increasing. Whether this increase is also due to the methods currently being used to assess kidney function in the elderly is still a matter of discussion. We aimed to describe the actual referral pattern of CKD patients in a large nephrology unit and test whether the use of different formulae to estimate kidney function could affect the staging and the need for specialist care in the older subset of our population. In 2019, 1992 patients were referred to our center. Almost 28% of the patients were aged ≥80 and about 6% were ≥90 years old. Among the causes of kidney disease, glomerulonephritis displayed a higher prevalence in younger patients whereas hypertensive or diabetic kidney disease were more prevalent in older patients. The prevalence of referred patients in advanced CKD stages increased with age; estimated glomerular filtration rate (eGFR) decreased with age regardless of which equation was used (chronic kidney disease epidemiology collaboration (CKD-EPI), Lund–Malmö Revised (LMR), modification of diet in renal disease (MDRD), Full Age Spectrum (FAS), or Berlin Initiative Study 1 (BIS)). With CKD-EPI as a reference, MDRD and FAS underestimated the CKD stage while LMR overestimated it. The BIS showed the highest heterogeneity. Considering an eGFR threshold limit of 45 mL/min for defining “significant” CKD in patients over 65 years of age, the variability in CKD staging was 10% no matter which equation was used. Our study quantified the weight of “old” and “old-old” patients on follow-up in a large nephrology outpatient unit and suggested that with the current referral pattern, the type of formula used does not affect the need for CKD care within the context of a relatively late referral, particularly in elderly patients.

## 1. Introduction

The aging of the world’s population, particularly in high-income countries, has changed the profile of several specialties. This is true of nephrology. In the current clinical setting, in western countries, the main causes of chronic kidney disease (CKD) have switched from glomerulonephritis, typical of the young, to nephroangiosclerosis or diabetic nephropathy and multifactorial diseases, typical of elderly individuals [1]. On the one hand, the current diagnosis and staging system of CKD underlines the importance of the early stages of CKD and, on the other hand, defines CKD as a long-lasting decrease in kidney function regardless of age [2]. The presence of proteinuria is an ancillary criterion. Globally, CKD is present in 8–15% of the world’s population, a prevalence that is intermediate between that of diabetes, a much better-acknowledged chronic, non-communicable disease, and that of hypertension, which reaches 50% in high-income, high life-expectancy settings [3].

The definition of CKD as an eGFR below 60 mL/min for at least 3 months has challenged, in particular, the geriatrists, raising the question of whether this definition should be adapted to age, acknowledging the para-physiological decrease that is observed in older individuals [4]. In fact, it has been proposed that we adapt our definition of CKD according to age groups, considering, for instance, an estimated glomerular filtration rate (eGFR) of 45 mL/min as a threshold in patients older than 65 years [5]. The question is not only semantic, and it reminds us of a similar discussion that regarded hypertension. While the definition of hypertension is not linked to age, the need for anti-hypertensive treatment has to be contextualized to age, to avoid the risks of over-zealous treatment and drug side effects [6]. Similarly, not all patients with lower eGFR should probably undergo costly and demanding follow-up.

Even though the kidney diseases of the elderly frequently progress more slowly compared to diseases occurring in younger individuals, they are not always “benign”, and the median age of patients starting dialysis has increased from less than 50 years in the 1980s to over 70 years at present, while the definition of “old” kidney transplant recipients has increased from “above 50” in the late 1980s to “above 70” in the new millennium [7,8,9,10].

As a result of an increasing prevalence of older patients, the definitions of “old” patients in nephrology, dialysis, and transplantation have changed over time, and several groups have been identified, although sometimes defined differently. These include the young-old (usually 60–69 years of age, but occasionally 60–74 years); the old-old (usually defined as being 70–79 years old); and the older-old or oldest-old, a category usually encompassing those who are over 80 years of age [11,12,13,14,15].

While these distinctions further add to the semantic conundrum, they indicate that “not all elderly patients are alike” and that the clinical and treatment problems faced by old patients deserve more-precise definition. One of the hot points in the discussion on CKD in the elderly resides in the problems of assessment of kidney function [5].

There are good reasons for monitoring kidney function in the elderly. First of all, correctly estimating kidney function is fundamental if drug toxicity is to be avoided, guiding choice as well as dosage; and secondly, timely interventions may reduce the need for renal replacement therapy and avoid the difficult dilemma of whether or not to start dialysis in the oldest-old, in whom the start of renal replacement therapy is associated with a high risk of rapid impairment in terms of clinical condition and quality of life [16,17,18].

Finding a balance between a minimalist approach (elderly patients are universally classified as having CKD; we cannot follow all elderly patients) and an interventionist one (all patients with CKD should be followed up regardless of age) is not simple, and the limited availability of nephrology care may further increase discrepancies in clinical management [19,20].

While the dialysis population is now monitored in most of the high-income countries and registries are being developed in medium-income settings, enabling the quantification of the burden of “elderly” patients on renal replacement therapy, less is known about the clinical burden and the main characteristics of elderly patients referred for nephrology care [21]. Such data are important for tailoring interventions and for better coordinating the limited medical resources available.

It is against this background that the present study was designed to contribute to filling a major knowledge gap, by analyzing the main clinical characteristics and kidney function of elderly patients referred to a large non-university hospital in France, a country where CKD is eligible for fully reimbursed care. We employed the most commonly used formulae to estimate eGFR to highlight their effect, if any, on the staging employed in identifying a need for nephrology care and to assess the prevalence of the different categories of “old patients” referred to our units for nephrology follow-up.

## 2. Materials and Methods

### 2.1. Setting of Study

The present study was undertaken at Centre Hospitalier Le Mans (CHM), one of the largest non-university hospitals in France. CHM has a nephrology service with a network of outpatient-care facilities (consultations and day-hospital) and is the only hospital in the department of Sarthe with nephrology beds (Sarthe: 560,227 inhabitants on 1 January 2020). The hospital is situated in the main city of the department, Le Mans, which has 143,325 inhabitants.

### 2.2. Characterization of Patients in the Cross-Sectional Analysis

All patients older than 18 who attended at least one consultation in 2019 in the nephrology outpatient clinics at CHM were included in the study. Patients on renal replacement therapy and kidney-transplant recipients were excluded. Patients’ data were retrieved from their electronic medical records (ORBIS). Kidney function was assessed by means of the following equations: chronic kidney disease-epidemiology collaboration (CKD-EPI), modification of diet in renal disease (MDRD), Lund—Malmö Revised (LMR), Full Age Spectrum (FAS), and Berlin Initiative Study 1 (BIS) [22,23,24,25,26]. Stratification was performed as per Kidney Disease: Improving Global Outcomes (KDIGO) guidelines, according to the presence of morphological abnormalities, urinary alterations, and kidney function. All patients considered to have CKD had at least two serum creatinine values, at least three months apart, or other signs of persistent kidney disease (proteinuria for at least three months, morphological abnormalities, etc.) as per KDIGO guidelines [2]. When more than one visit in 2019 was present in the medical records, CKD stage was defined according to the last serum creatinine value. Patients in a phase of evaluation or with a single serum creatinine value available were considered to be “missing stages”. Since all patients were outpatients, the incidence of acute kidney injury (AKI) was considered to be negligible, unless explicitly mentioned in the last clinical consultation report.

### 2.3. Data Gathered

The following data were gathered: demographic characteristics (gender and age) and type of kidney disease. Causes of kidney disease were classified into 10 categories (glomerulonephritis, nephroangiosclerosis/hypertensive nephropathy, diabetic kidney disease, congenital anomalies of the kidney and urinary tract (CAKUT)/obstructive/systemic disease/solitary kidney, polycystic kidney disease (ADPKD), isolated urinary abnormalities, multifactorial, postpartum-preeclampsia, renal stones, and other/not known/post-acute kidney injury (AKI)) in accordance with the diagnosis made by the attending nephrologist based on the patient’s medical history, the availability of a histologic assessment (kidney biopsy), and his/her expertise. All diagnoses were reviewed and confirmed by the senior nephrologist (G.B.P.).

### 2.4. Statistical Analysis

Statistical analyses were performed using SPSS Statistics version 23 (IBM Corp., Armonk, NY, USA). Quantitative data were expressed as median values (min–max), and qualitative data were presented as proportions and percentages.

The normality and homoscedasticity hypotheses were tested with the Shapiro–Wilk and Levene’s tests, respectively, for continuous series. The Student’s *t*-test was performed to compare two non-paired groups, otherwise the Wilcoxon rank sum test was used. Variance analysis was applied for additional group comparisons, otherwise the Kruskal–Wallis test was performed. Proportions were tested using the Chi-square or the Fisher exact test in case of a low subsample cohort size (<5). A two-sided alpha risk was set at 5%.

### 2.5. Ethical Issues

The study was conducted in accordance with the Declaration of Helsinki. The cross-sectional observational study involved the analysis of the clinical charts of patients who attended at least one consultation in a nephrology outpatient clinic in 2019; the anonymized database was built following the requirements of the regional health council, to assess the number of cases in CKD stages 4 and 5. The study was approved by the CHM ethical committee at its 24 September 2020 meeting.

## 3. Results

### 3.1. Baseline Data

The demographic characteristics of the cohort of 1992 patients referred for at least one consultation to Centre Hospitalier Le Mans are reported in Table 1.

The highest prevalence was recorded in the 80- to 89-year-old age group (21.9%) while the oldest-old, aged 90 or older, accounted for 5.8% of the referred cases (Table 1).

In the context of this relatively old population, chronic kidney diseases varied significantly between age groups. While the prevalence of glomerulonephritis declined with age, the prevalence of three categories increased sharply. Multifactorial disease, nephroangiosclerosis, and kidney diseases associated with diabetes accounted for only 12.7% of the cases in the younger age group but for 80.7% in the 80- to 89-year-old age group and 84.6% in patients aged 90 or older (Table 1).

In keeping with a high prevalence of vascular nephropathies, which are usually non-proteinuric or characterized by mild proteinuria, the prevalence of the cases that displayed proteinuria over 1 g per day was low. It is worth noting that proteinuria was missing in several cases; the lack of regular control is partly a reflection of the fact that proteinuria had not been regularly checked in patients who were classified as having a vascular disease, and were known to have low or absent proteinuria in previous tests (Figure S1).

Most kidney diseases were present in all age groups; the relatively high prevalence of cases with lithiasis or postpartum preeclampsia reflects specific referral patterns developed in the study center: for instance, younger patients with lithiasis are referred to the nephrologist for evaluation due to the higher probability of a genetic disease or a tubule-interstitial disorder, while older patients are usually followed up by urologists in the absence of an impairment of kidney function.

### 3.2. Kidney Function Data

In the population on follow-up, median serum creatinine steadily increased, and median eGFR consequently decreased with age, regardless of the formula employed (Table 1 and Table 2).

**Figure 1:**
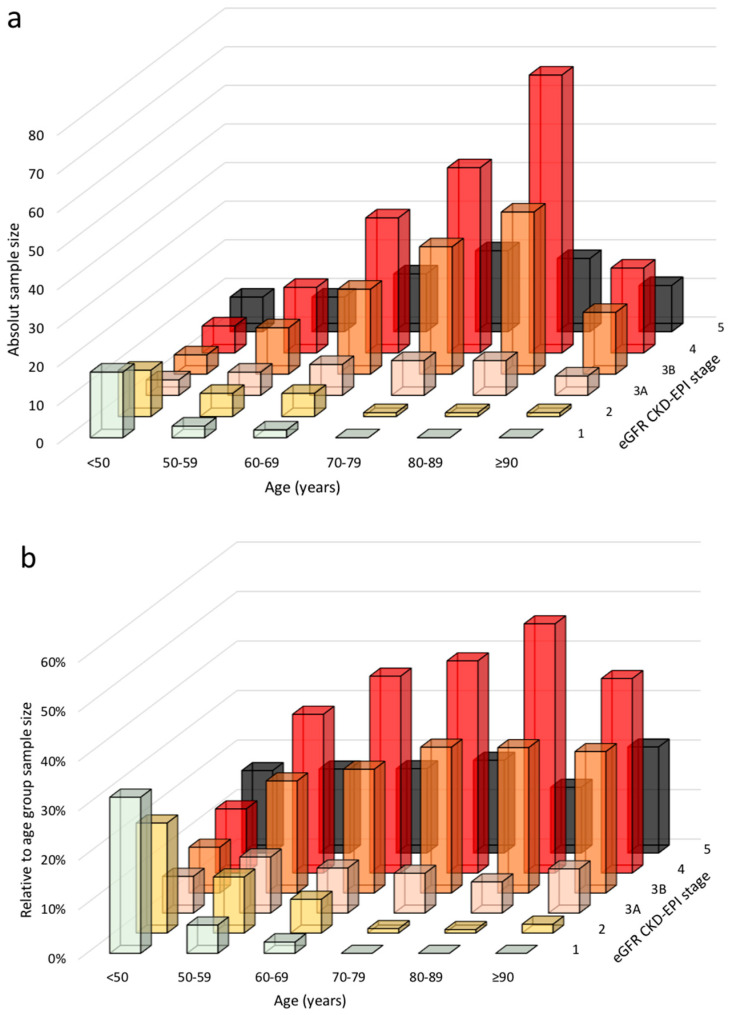
Distribution of chronic kidney disease-epidemiology collaboration (CKD-EPI) stages by age group in patients followed up by the Centre Hospitalier Le Mans (CHM) nephrology outpatient units: (**a**) absolute numbers, (**b**) relative number inside the age group. eGFR: estimated glomerular filtration rate.

The highest median level of serum creatinine corresponded to 1.88 mg/dL at or above age 90. In line with these observations, the prevalence of CKD stages 4 and 5 increased. Only 1.7% of the patients aged 90 or older had a CKD stage of 1 or 2; over 60% of those in this age group were in CKD stages 4–5, versus 38.5% in the 80- to 89-year-old age group and only 8.3% of patients aged under 50 (Table 2 and Figure 1).

The distribution of stages was different in different age groups (Figure 1). The late CKD stages were more often found in the old-old and extremely old patients, suggesting a selective referral of more impaired kidney function with advanced age (Figures S1 and S2).

**Figure 2:**
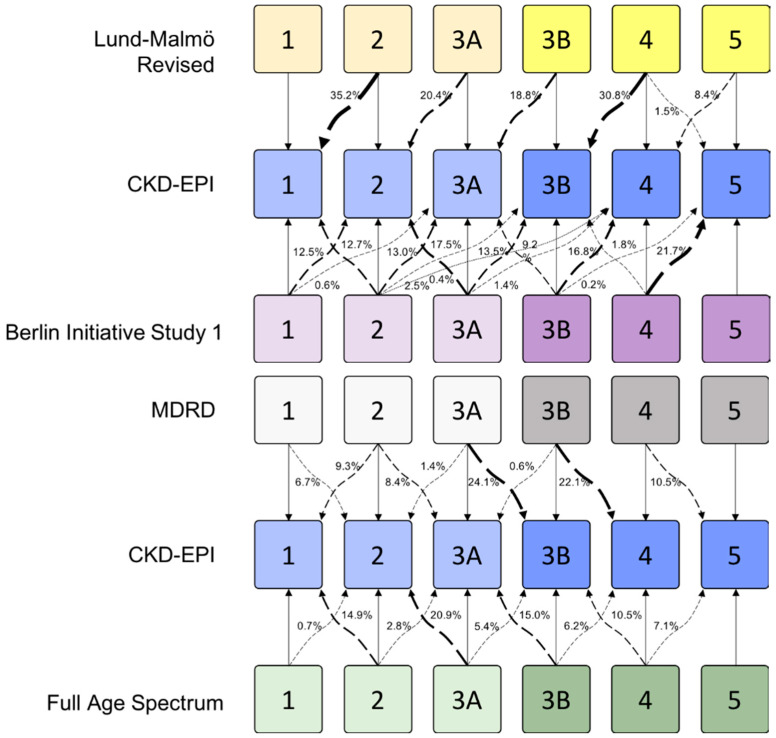
Percentage of patients changing CKD stage based on comparisons of the results from other formulae with CKD-EPI. Curved arrows show the percentage of cases that changed classification according to the formula used. Straight arrows indicate the remaining cases that did not change stage.

**Figure 3:**
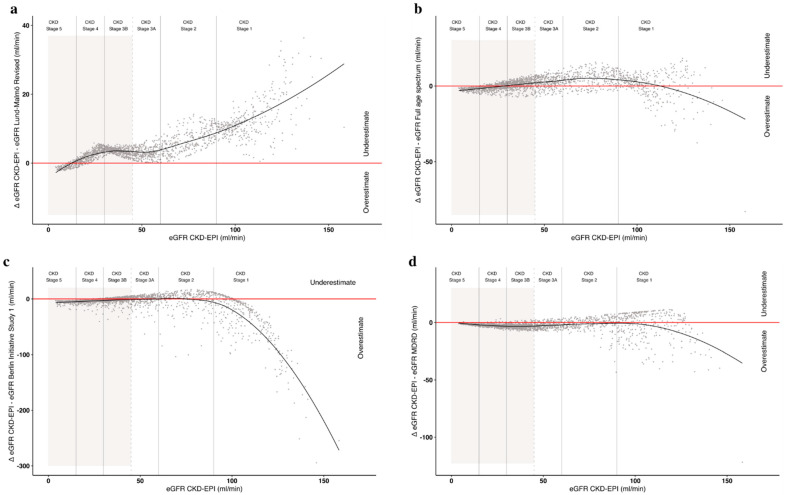
Estimated glomerular filtration rate (eGFR) overestimation or underestimation according to different formulae compared to CKD-EPI in our cohort: (**a**) Lund–Malmö Revised; (**b**) Full Age Spectrum; (**c**) Berlin Initiative Study 1; and (**d**) modification of diet in renal disease (MDRD). The grey background identifies a glomerular filtration rate ≤ 45 mL/min.

In the context of good correlations between the most widely used formula (CKD-EPI); the classic MDRD formula; and other, more recent formulae targeted toward elderly patients, there were some differences in the results from the different equations (Table 3, Table S1, Figure 2 and Figure 3). The highest heterogeneity was observed with the Berlin Initiative Study 1 formula (Figure 2), while the Lund–Malmö Revised tended to overrate CKD stages (Figure 2) and underestimate eGFR (Figure 3) in opposition to the MDRD. By contrast, the Full Age Spectrum tended to classify patients with advanced kidney disease in lower CKD stages compared to the reference CKD-EPI (Figure 2).

However, if we consider two broad groups of eGFR (dichotomized at 45 mL/min based on the most recent debate in the literature [5]) in the population followed up by the CHM nephrology outpatient units, the overall differences are below 10% in the entire study population and are less relevant in the old-old and oldest-old patients than in the younger subset of cases (Table 3, Table S1).

## 4. Discussion

The analysis of this large cohort of about 2000 patients clearly shows that nephrologists need geriatric competencies; although the hospital has two dedicated outpatient units for the treatment of complicated lithiasis and postpartum-preeclampsia follow-up of mainly young patients, the vast majority of patients followed up in the setting of this study (a large non-university hospital in central France) were classifiable as “old” (Table 1, Figure 1).

Patients aged 60 or older accounted for about 70% of the overall cohort, and about one patient out of four was aged 80 or older (“old-old”); the most numerous subset was recorded in the 80- to 89-year-old age group (21.9%), while the extremely old patients, aged 90 or older, accounted for 5.8% of the cases (Table 1).

As for kidney diseases, the prevalence of multifactorial diseases, nephroangiosclerosis, and diabetes-associated kidney disease, particularly the variant with low proteinuria (diabetes-vascular), increased with age. These three diseases accounted for over 80% of the diagnoses in patients aged 80 or older at the time of our study (Table 1). The diagnoses of vascular kidney diseases, including nephroangiosclerosis, are eminently clinical (hypertension, signs of central or peripheral vascular disease, small kidneys, absent or scant proteinuria, and no evidence of a different kidney disease), and may also reflect a minimalist approach toward diagnosing kidney diseases in the elderly. However, their prevalence in younger patients is in line with international standards, and this should reassure us that diagnostic minimalism is not widespread in our setting [27].

Serum creatinine increased steadily with age in the study cohort (Table 1). As a consequence, regardless of the formula used to assess eGFR, the estimated glomerular filtration rate decreased with age, with a consequent increase in the prevalence of CKD stages 4 and 5. Taking the results obtained using the CKD-EPI equation as a reference, only 1.7% of the patients on follow-up aged 90 or older had normal or slightly reduced kidney function, and over 60% of these cases were in CKD stage 4–5. In the 80–89 age group, 38.5% of the patients were in stage 4–5, while only 8.3% of the patients aged less than 50 were in advanced CKD stages (Figure 3). The use of different formulae did not substantially change the overall picture (Figure 3).

This distribution disproves the frequently reported concern that since the commonly used formulae are less reliable in the elderly, elderly patients are being over-referred, at levels of kidney function that do not represent “true kidney disease”. Hence, some authors suggest that the definition of CKD should start from <45 mL/min of eGFR in the elderly [5]. Of note, in the presently referred cohort, less than 10% of patients would have been shifted backwards, and this bias is less frequently observed in the elderly (Table 3).

While this present cross-sectional study did not allow us to analyze trajectories, there is little doubt that patients in CKD stages 4 and 5 are more fragile and prone to developing severe metabolic derangements; have a higher risk of death and a greater need to start renal replacement therapy; and should, therefore, be promptly identified [12,28]. The subset of cases with eGFR < 30 mL/min increased sharply with age, thus raising more concern about late referral than about an overzealous and possibly not cost-effective use of nephrology resources.

A strength of this study is that it is the only one recently carried out with the aim of assessing the burden of old, old-old, and extremely old patients followed up by nephrology units at a large general hospital. However, it has several limitations. First, it is a cross-sectional analysis, limited to one year only and without outcome data; second, even if the large majority of the patients had at least two data values for serum creatinine, thus allowing a diagnosis of CKD to be made, we considered only the latter value and did not analyze the frequency of variations during the year; and third, patients were followed up by five different nephrologists in two different macro-areas (one in general nephrology and one dedicated to the care of advanced CKD), and we did not attempt further analyses, for example, based on treatment or comorbidity. These points will be the subject of an in-depth analysis of the subset of patients more homogeneously followed up in the patient unit dedicated to advanced CKD stages.

Notwithstanding these limitations, this first portrait of the elderly population followed up by a large nephrology unit suggests that elderly patients are not over-referred and that, on the contrary, they may even be under-referred, thus limiting the efficacy of actions aimed at preventing CKD progression and the start of dialysis. Furthermore, with the current relatively late recruitment, the choice of the formulae used only minimally affected the prevalence of patients with less than 45 mL/min of eGFR, and the differences among formulae were lower in “old” and “old-old” patients. This should serve to reassure us that, in contrast to large-population studies, the information used in daily practice is not subject to drastic changes according to the eGFR formula employed.

## 5. Conclusions

Our study found that in a large nephrology outpatient unit a very high percentage of the patients were elderly, with a high prevalence of old-old and oldest-old cases. The current referral pattern is characterized by referrals of older patients with lower eGFR. In this setting, the use of different formulae does not lead to a relevant difference between patients with eGFR above or below 45 mL/min, the threshold now suggested for the definition of CKD in the elderly.

## Figures and Tables

**Table 1:** Baseline data: age and kidney diseases in the patient cohort followed up by the Centre Hospitalier Le Mans (CHM) nephrology outpatient units in 2019.

	Age Groups						
	<50	50–59	60–69	70–79	80–89	≥90	*p*-Values
**N (total: 1992)**	379	216	414	431	436	116	
**Males/females**	154/225	114/102	263/151	302/129	245/191	56/60	**<0.001**
**Creatinine (mg/dL), median (IQR)**	0.85 (0.48)	1.09 (0.95)	1.39 (0.94)	1.65 (1.07)	1.67 (0.84)	1.88 (1.13)	**<0.001**
**eGFR EPI (mL/min/1.73 m^2^), median (IQR)**	100 (47)	66 (58)	47 (35)	38 (27)	33 (17)	27 (18)	**<0.001**
**Proteinuria (g/L), *n* (%)**							0.147
<0.3	213 (69.4%)	119 (65.0%)	212 (60.7%)	240 (65.0%)	239 (62.3%)	67 (64.4%)	
0.3–1	59 (19.2%)	29 (15.8%)	83 (23.5%)	53 (14.4%)	93 (24.5%)	25 (24.0%)	
≥1	35 (11.4%)	35 (19.1%)	55 (15.8%)	76 (20.6%)	50 (3.2%)	12 (11.5%)	
**CKD stage according to CKD-EPI, *n* (%)**							**<0.001**
1	226 (62.3%)	63 (29.4%)	38 (9.3%)	10 (2.3%)	1 (0.2%)	0 (0%)	
2	58 (16%)	51 (23.8%)	96 (23.6%)	57 (13.4%)	27 (6.2%)	2 (1.7%)	
3A	27 (7.4%)	36 (16.8%)	86 (21.1%)	84 (19.7%)	60 (13.8%)	7 (6%)	
3B	22 (6.1%)	23 (10.7%)	98 (24.1%)	129 (30.3%)	180 (41.3%)	37 (31.9%)	
4	14 (3.9%)	23 (10.7%)	60 (14.7%)	110 (25.8%)	131 (30%)	53 (45.7%)	
5	16 (4.4%)	18 (8.4%)	29 (7.1%)	36 (8.5%)	37 (8.5%)	17 (14.7%)	
**Main diagnosis of kidney disease**							**<0.001**
Glomerulonephritis	52 (13.7%)	22 (10.2%)	30 (7.2%)	22 (5.1%)	10 (2.3%)	3 (2.6%)	
Nephroangiosclerosis/hypertensive nephropathy	13 (3.4%)	15 (6.9%)	60 (14.5%)	107 (24.8%)	205 (47%)	70 (60.3%)	
Diabetic kidney disease	17 (4.5%)	29 (13.4%)	98 (23.7%)	109 (25.3%)	85 (19.5%)	16 (13.8%)	
CAKUT/obstructive/systemic disease/solitary kidney	39 (10.3%)	16 (7.4%)	27 (6.5%)	35 (8.1%)	24 (5.5%)	7 (6%)	
ADPKD	25 (6.6%)	17 (7.9%)	18 (4.3%)	7 (1.6%)	8 (1.8%)	0 (0%)	
Isolated urinary abnormalities	12 (3.2%)	4 (1.9%)	6 (1.4%)	4 (0.9%)	1 (0.2%)	0 (0%)	
Multifactorial	25 (6.6%)	27 (12.5%)	70 (16.9%)	78 (18.1%)	80 (18.3%)	13 (11.2%)	
Other/post AKI/not known	29 (7.6%)	15 (6.9%)	24 (5.7%)	26 (6.1%)	12 (2.7%)	6 (5.2%)	
Postpartum-preeclampsia	68 (17.9%)	0 (0%)	0 (0%)	0 (0%)	0 (0%)	0 (0%)	
Renal stones	99 (26.1%)	71 (32.9%)	81 (19.6%)	43 (10%)	11 (2.5%)	1 (0.9%)	

IQR: Inter-quartile range; ADPKD: autosomal dominant polycystic kidney disease; CAKUT: congenital anomalies of the kidney and urinary tract; AKI: acute kidney injury; and eGFR-EPI: estimated glomerular filtration rate according to the chronic kidney disease-epidemiology collaboration (CKD-EPI) equation.

**Table 2:** Baseline data: kidney function in the patient cohort followed up by the CHM nephrology outpatient units in 2019.

	Age Groups					
	<50	50–59	60–69	70–79	80–89	≥90
N (total = 1992)	379	216	414	431	436	116
Creatinine (mg/L), median (IQR)	0.85 (0.48)	1.09 (0.95)	1.39 (0.94)	1.65 (1.07)	1.67 (0.84)	1.88 (1.13)
eGFR (mL/min/1.73 m^2^), median (IQR)						
CKD-EPI	100 (47)	66 (58)	47 (35)	38 (27)	33 (17)	27 (18)
Lund–Malmö Revised	88 (34)	64 (51)	45 (35)	34 (27)	29 (17)	22 (15)
Full age spectrum	96 (41)	65 (49)	46 (29)	36 (22)	31 (14)	25 (13)
Berlin Initiative Study 1	125 (81)	69 (47)	49 (27)	39 (20)	33 (13)	27 (12)
MDRD	92 (47)	65 (54)	49 (35)	41 (28)	37 (19)	32 (20)

IQR: Inter-quartile range. MDRD: Modification of Diet in Renal Disease formula.

**Table 3:** Proportion of CKD-stage changes across the 45 mL/min threshold according to estimated glomerular filtration rate (eGFR) estimation formula by age group in patients on follow-up at the CHM nephrology outpatient units.

	eGFR Estimation Formula				
	CKD-EPI	Lund–Malmö Revised	Full Age Spectrum	Berlin Initiative Study 1	MDRD
	**Overall**				
% vs. CKD-EPI	0	7.2%	5.86%	0.1%	−8.52%
*N*	1044	1125	1109	1045	962
	**<60 years of age**				
% vs. CKD-EPI	0	7.09%	−13.46%	−45.68%	1.67%
*N*	118	127	104	81	120
	**≥60 to <80 years of age**				
% vs. CKD-EPI	0	6.96%	5.84%	−1.3%	−8.84%
*N*	468	503	497	462	430
	**≥80 years of age**				
% vs. CKD-EPI	0	7.47%	9.84%	8.76%	−11.17%
*N*	458	495	508	502	412

## Data Availability

The data presented in this study are available on request from the corresponding author.

## Supplementary Materials

The following are available online at https://www.mdpi.com/2077-0383/10/6/1168/s1, Figure S1: Distribution of classes of proteinuria by age groups. Figure S2: Distribution of the CKD-EPI equation stages by age groups. Table S1: Proportion of CKD-stage changes according to eGFR estimation formula by age category.
