# Supplementary material for: Elderly Patients in a Large Nephrology Unit: Who Are Our Old, Old-Old and Oldest-Old Patients?
Source: J Clin Med. 2021 Mar 11;10(6):1168. doi: 10.3390/jcm10061168 (PMC8000250; doi:10.3390/jcm10061168)
Supplement: Supplementary file 1 [file jcm-10-01168-s001.pdf]

Supplementary material

Figure S1. Distribution of classes of proteinuria by age groups.

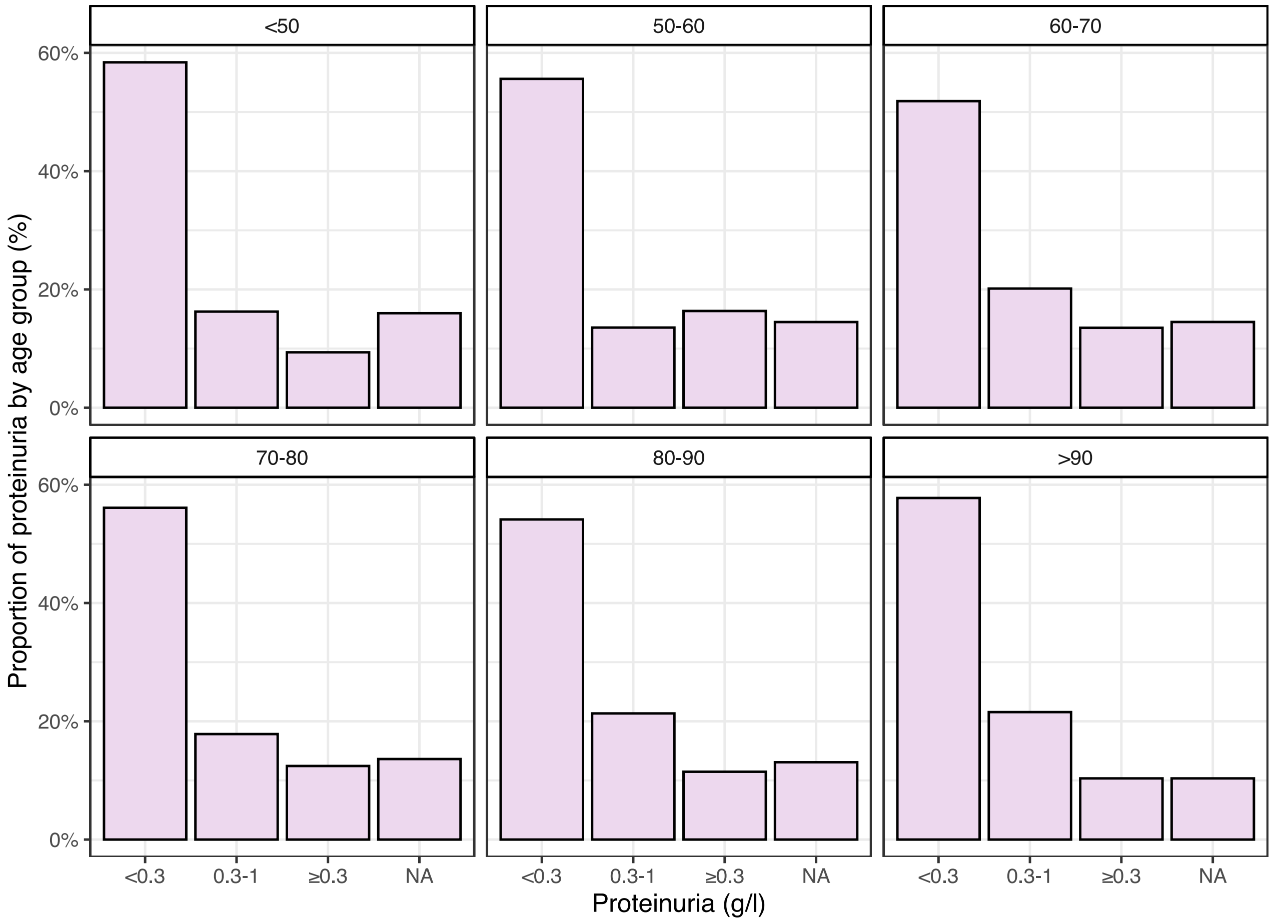

Legend NA: Not available.

Figure S2. Distribution of the CKD-EPI equation stages by age groups.

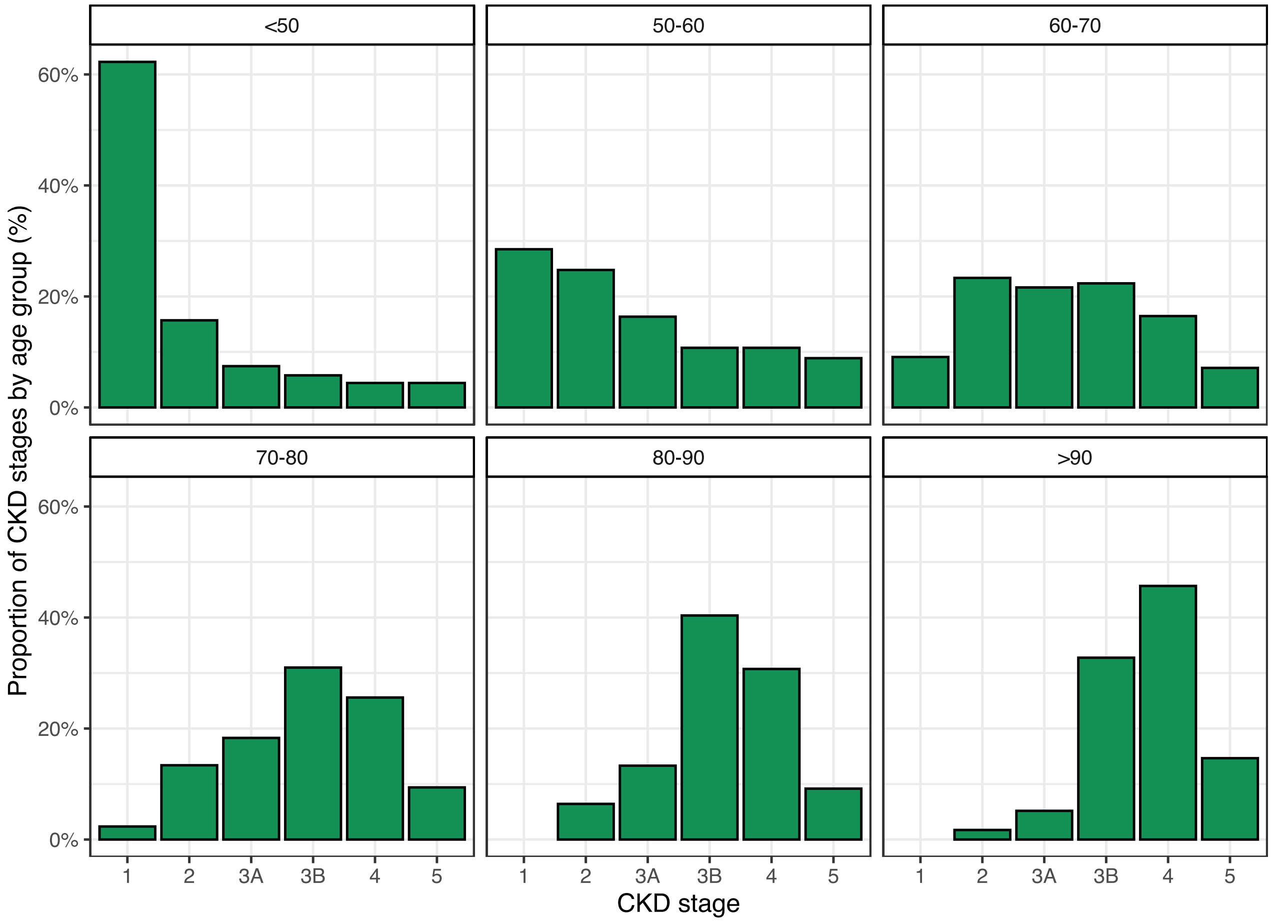

Legend: CKD: chronic kidney disease.

Table S1. Proportion of CKD-stage changes according to eGFR estimation formula by age category.

Stages		eGFR estimation formula CKD-EPI	Lund-Malmö Revised	Full Age Spectrum	Berlin Initiative Study 1	MDRD
				**Overall**		
**1 to 3A**	% vs. CKD-EPI	0	-9.7%	-7.6%	-0.1%	8.2%
	n	918	837	853	917	1000
**3B to 5**	% vs. CKD-EPI	0	7.2%	5.9%	0.1%	-8.5%
	n	1044	1125	1109	1045	962
**4 to 5**	% vs. CKD-EPI	0	19.0%	2.4%	-20.6%	-25.1%
	N	563	695	577	467	450
				**<60 years of age**		
**1 to 3A**	% vs. CKD-EPI	0	-2.0%	3.0%	7.5%	-0.4%
	n	459	450	473	496	457
**3B to 5**	% vs. CKD-EPI	0	7.1%	-13.5%	-45.7%	1.7%
	n	118	127	104	81	120
**4 to 5**	% vs. CKD-EPI	0	10.8%	-19.4%	-60.9%	-2.8%
	n	74	83	62	46	72
				**<80 to ≥60 years of age**		
**1 to 3A**	% vs. CKD-EPI	0	-10.6%	-8.6%	1.6%	9.4%
	n	365	330	336	371	403
**3B to 5**	% vs. CKD-EPI	0	7.0%	5.8%	-1.3%	-8.8%
	n	468	503	497	462	430
**4 to 5**	% vs. CKD-EPI	0	16.4%	-8.9%	-33.2%	-22.0%
	n	245	293	225	184	201
				**≥80 years of age**		
**1 to 3A**	% vs. CKD-EPI	0	-64.9%	-113.6%	-88.0%	32.9%
	n	94	57	44	50	140
**3B to 5**	% vs. CKD-EPI	0	7.5%	9.8%	8.8%	-11.2%
	n	458	495	508	502	412
**4 to 5**	% vs. CKD-EPI	0	23.5%	15.9%	-3.0%	-37.9%
	n	244	319	290	237	177
